# Oral Hygiene Habits of Complete Denture Wearers in Central Transylvania, Romania

**DOI:** 10.3290/j.ohpd.b965699

**Published:** 2021-02-19

**Authors:** Cecilia Bacali, Vivi Nastase, Mariana Constantiniuc, Liana Lascu, Mandra Eugenia Badea

**Affiliations:** a Lecturer and Dentist, Department of Prosthodontics and Dental Materials, Faculty of Dental Medicine, University of Medicine and Pharmacy “Iuliu Hatieganu”, Cluj-Napoca, Romania. Idea, hypothesis, experimental design, gathered and analysed the data in partial fulfillment of requirements for the PhD degree, wrote the manuscript.; b Researcher, Institute for Computational Linguistics, University of Heidelberg, Heidelberg, Germany. Proofread the manuscript, consulted on and performed statistical analyses.; c Professor, Department of Prosthodontics and Dental Materials, Faculty of Dental Medicine, University of Medicine and Pharmacy “Iuliu Hatieganu”, Cluj-Napoca, Romania. Study design, proofread the manuscript.; d Professor, Department of Prosthodontics and Dental Materials, Faculty of Dental Medicine, University of Medicine and Pharmacy “Iuliu Hatieganu”, Cluj-Napoca, Romania. contributed to the discussion, proofread the manuscript.; e Professor, Department of Preventive Dental Medicine, Faculty of Dental Medicine, University of Medicine and Pharmacy “Iuliu Hatieganu”, Cluj-Napoca, Romania. Idea, study design, proofread the manuscript.

**Keywords:** complete dentures, denture cleaners, oral hygiene habits, nocturnal wear, stomatitis

## Abstract

**Purpose:**

To identify the oral hygiene habits and denture hygiene status (e.g. sociodemographic characteristics, general health status) of complete denture wearers in Central Transylvania, Romania.

**Materials and Methods:**

This descriptive cross-sectional study was based on an original self-administered questionnaire. It included 162 patients who experienced complete tooth loss in one or both jaws and wore polymethylmethacrylate acrylic (PMMA) complete dentures. The exclusion criteria were partial dentures, dentures fabricated from materials other than PMMA, and the patient being unable to provide all the required information. The questionnaire solicited information regarding sociodemographic status, general health and oral hygiene-related habits. The dentures were clinically evaluated in order to assess denture hygiene.

**Results:**

Most respondents were completely edentulous (38.0% wore maxillary dentures, 10.6% mandibular dentures, while 51.6% had both), with an average age above 60, all wearing dentures. The clinical evaluation of the dentures revealed that 12.3% exhibited optimal hygiene status, 40.1% good, 32.7% unsatisfactory and 14.8% bad, with more women than men having well-cleaned dentures. Cleaning frequency was found to be 2–3 times per day for 54.3% of the patients, with 93.2% of the subjects using a toothbrush and 76.5% using toothpaste. Most of the participants felt at ease with the cleaning procedures. Only 30.9% of the respondents reported denture removal overnight. The results showed that the hygiene of the patients’ dentures was not correlated with their answers regarding oral hygiene habits.

**Conclusions:**

Competent oral health and denture hygiene promotion should be established, especially regarding nocturnal denture removal, denture hygiene methods, instruments and cleaning frequency.

Evidence of toothbrush use for oral hygiene dates back to ancient Babylonia. Toothpaste and mouthwash were also invented in ancient times, although their formulas have improved significantly since then.^24^ Still, medical conditions associated with poor oral hygiene, such as dental caries, loss of periodontal support and tooth loss, are still highly prevalent. In patients with removable dentures, oral mucosal lesions may be reactions to microbial denture plaque resulting from poor hygiene, denture material or denture-inflicted mechanical injury. The most prevalent oral lesions associated with the wearing of removable dentures are denture stomatitis (50%), angular cheilitis (15%), traumatic ulcers (5%), denture irritation hyperplasia (12%) and flabby ridges (10-20%). Chronic lesions of the oral mucosa can lead to more severe diseases, such as oral carcinoma.^7^ A survey of epidemiological studies found that between 15% to 70% of denture wearers suffer from denture stomatitis caused by poor oral hygiene or wearing dentures overnight.^18^ Besides the correlation between oral hygiene and oral mucosal lesions, many studies have also shown a link between poor oral hygiene and systemic conditions, such as aspiration pneumonia, endocarditis or obstructive pneumonia. Given the magnitude of the impact on general health, the World Health Organization has declared oral health a public health issue.^33,34^

Oral health care in denture wearers is particularly important for overall health, especially in the elderly. Regular cleaning of dentures is considered an important part of oral hygiene for denture wearers. Lack of denture hygiene is one of the main etiological factors causing the inflammation of edentulous patients’ oral mucosa.^1^ For denture wearers, an appropriate oral hygiene depends on several variables related to the patient, dentist and health system, and also to the denture material, as well as oral hygiene products and techniques. Factors such as lack of knowledge, not understanding the technique, lack of interest and motivation or factors caused by aging depression, memory loss, movement impairment and other sociodemographic variables can impact oral hygiene in denture wearers.^25,41^ Inadequate oral hygiene practices or overnight denture wearing are factors that are linked to oral inflammations and even pneumonia.^16,21,27^

Among the most effective and commonly used denture cleaning techniques are denture brushing and immersion in disinfectant solutions.^22^ While each of them is effective separately, their combination leads to the best results.^32,37^ Microwave disinfection of dentures is considered an attractive alternative to the use of chemicals, with similar or even superior results.^23^ Combined educational programmes where specialists instruct caregivers in denture cleaning techniques and ultrasound technologies for denture cleaning may have beneficial effects on oral health in assisted seniors.^43^

With respect to the impact of denture base material on denture cleaning, acrylic dentures present surface irregularities and micropores that favour bacterial adhesion and colonisation.^22^ Over time, acrylic dentures suffer an aging process allowing bacteria to develop, making it highly recommendable to occasionally repair or replace old dentures.^22,38^

The aim of the present study was to identify the sociodemographic characteristics and general health status that can influence oral hygiene habits, and to assess the denture hygiene status of complete denture wearers in Central Transylvania.

## Materials and Methods

This study was based on a descriptive, cross-sectional survey used a self-administered original questionnaire, approved by the ethics committee of the “Iuliu Hatieganu” University of Medicine and Pharmacy, Cluj-Napoca (nr. 428/24.11.2016). The questionnaire was pre-tested on 30 denture wearers who volunteered (data gathered were not included in the study), and then validated. The questionnaires were distributed to complete denture wearers selected randomly during their dental appointments at the Department of Prosthodontics and Dental Materials of the Faculty of Dental Medicine Cluj-Napoca and different dental offices across Central Transylvania (Cluj, Mures, Salaj), in both urban and rural areas, over a six-month period. The purpose of the survey was explained to the participants and written informed consent was obtained. Participation was voluntary and anonymous; all measures were taken to ensure confidentiality. There was no time limit for filling in the questionnaire. Participation criteria consisted of total tooth loss on one or both jaws and complete polymethyl methacrylate dentures. Exclusion criteria were: partial tooth loss/partial dentures, dentures fabricated from other materials such as polyamide, and the patient being unable to provide all the required information.

The questionnaire included general questions regarding sociodemographic status such as age, gender, activity and residence, followed by smoking habits, general health status, main cause of tooth loss and specific questions regarding oral hygiene habits.

During the dental visit, a clinical evaluation of denture hygiene was performed, using the Budtz-Jörgensen denture plaque index.^6^ The assessment criteria were optimal, good, unsatisfactory, and bad, based on 4 grades of plaque extension on the internal (mucosal) surface of the denture: 0 = nonvisible, 1 = less than one-third, 2 = one-third to two-thirds, 3 = more than two-thirds. A staining of the denture internal surface with methylene blue, using a syringe, was used in order to facilitate assessment.

The primary outcome variables were the denture hygiene status and dentures on one or both jaws, in one set of analysis, and the oral hygiene habits in another. The secondary variables were oral hygiene habits (with respect to denture hygiene status) and sociodemographic characteristics and general health (with respect to oral hygiene habits).

Categorical data were presented as number (n) and percentages, along with 95% confidence intervals, computed with the Clopper and Pearson method for binary data^10^ and with the Sison and Glaz method for nominal data.^40^ The association between two categorical variables was assessed with the chi-squared or Fisher’s exact test (if expected frequencies were below 5 in more than 20% of the cells). For all statistical tests, a 95% confidence level and two tailed p-values were used. All analyses were performed using the R environment for statistical computing and graphics, version 3.4.3 (https://www.r-project.org).

## Results

The information elicited through the questionnaire and the profile of the study group are presented in Table 1. 162 patients completed the questionnaire, of which 65.4% were women; 69.1% of the respondents were above 61 years of age, 59.0% were urban residents and 77.1% were retired from activity; 79.6% of the patients reported associated diseases, the most frequent being cardiovascular disorders; 24.7% of the patients were current smokers.

**Table 1:** Description of sociodemographic characteristics, health status and denture-related information

Variable	n	%
Total	n=162	100
**Age group**		
<40 years	2	1.23
41–50 years	18	11.11
51–60 years	30	18.52
61–70 years	54	33.33
>71 years	58	35.8
**Gender**		
Male	56	34.57
Female	106	65.43
**Occupation**		
Employed	37	22.84
Retired	125	77.16
**Education**		
Primary school	29	17.9
Secondary school	50	30.86
High school	67	41.36
University	16	9.88
**Residence**		
Urban area	95	58.64
Rural area	67	41.36
**Health status**		
Cardiovascular disease	83	51.23
Liver disease	7	4.32
Kidney disease	13	8.02
Gastrointestinal disease	21	12.96
Endocrine disease	19	11.73
Bone disease	38	23.46
Dermatological disease	8	4.94
Allergies	17	10.49
Other	7	4.32
Smoker	40	24.69
**Type of denture**		
Maxillary	61	37.89
Mandibular	17	10.56
Both	83	51.55
**Tooth-loss etiology**		
Caries	132	81.48
Parodontopathy	66	40.74
Injury	3	1.85

n=number of patients.

More than half of the participants were completely edentulous and wore both maxillary and mandibular dentures. 81.48% reported dental caries as the main cause of tooth loss.

**Fig 1:**
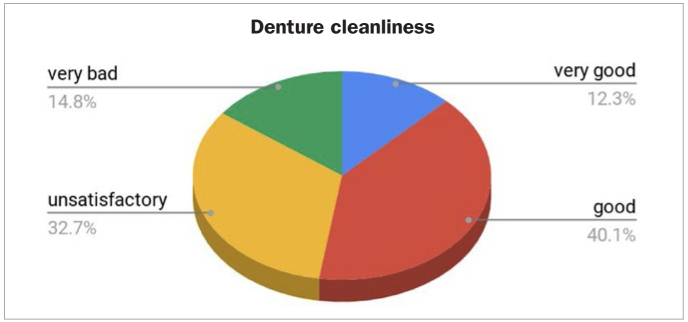
Denture hygiene status.

Denture hygiene status, assessed through clinical examination using the Budtz-Jörgensen denture plaque index^6^ on the mucosal surface, revealed that the hygiene was optimal for 12.3% of the dentures, good for 40.1%, unsatisfactory for 32.7%, while 14.8% showed bad denture hygiene, as shown in Fig 1.

**Fig 2:**
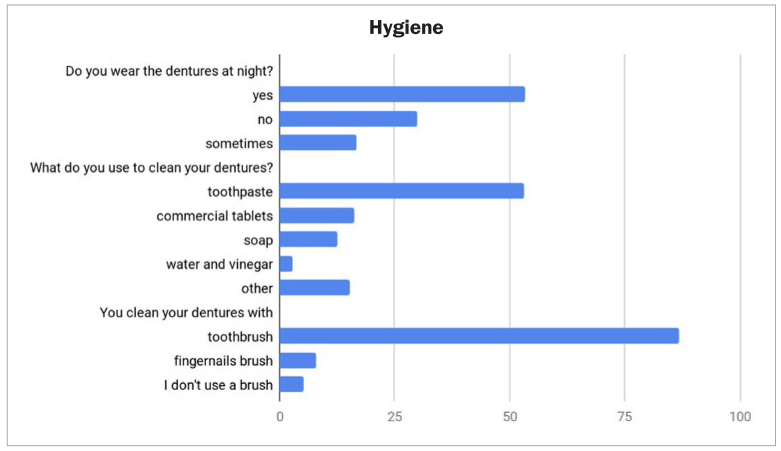
Denture cleaning habits of the respondents.

Cleaning procedures were considered easy by 94.4% of the questioned patients; 54.3% of the respondents reported cleaning their dentures 2-3 times a day, 40.1% of them once a day, while 5.6% once every other day. Regarding the methods, instruments and cleaning products used for denture hygiene (Fig 2), most of the respondents reported the use of a toothbrush (93.2%) and toothpaste (76.5%). 22.2% of the patients used commercial cleaning tablets, 17.3% used soap and 3.7% reported soaking the dentures into a water vinegar mix. Most of the patients wore their dentures overnight (52.5%).

**Fig 3:**
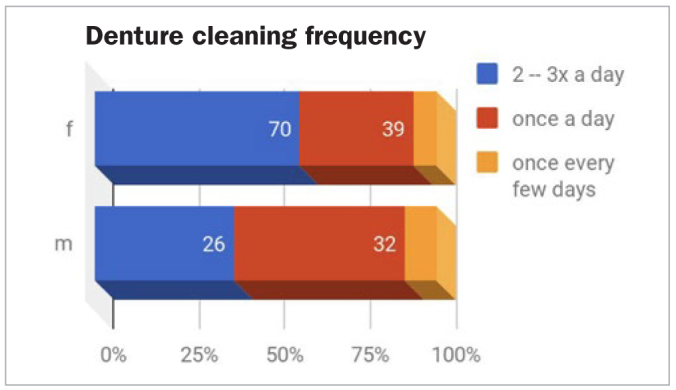
Denture cleaning frequency by gender (f, m).

A statistically significant difference (p = 0.048) between cleaning frequency by gender was found, as most of the women (61.3%) cleaned their denture twice per day, while 51.8% of men did so only once (Fig 3).

The impact of each of the sociodemographic and health variables on the hygiene habits and the denture hygiene status was investigated, but apart from gender, no associations were found.

## Discussion

Globally, the percentage of the elderly population is constantly growing. In Romania, life expectancy increased from 65 years for women and 61.5 for men in 1956 to 77.5 years for women and 70.1 years for men in 2011. This also results in an increase in several age-specific conditions, including oral health-related problems such as tooth loss, are common. Most of the elderly in Romania are affected by edentulism with high impact on physical, psychological and social dimensions.^28^ In 2003, a WHO report estimated that 26% of Romanians ages 65 to 74 years were edentulous.^34^ Despite progress in materials and methods used in dental treatment, extractions caused by preventable diseases such as dental caries and periodontal diseases are still frequent, mainly for economic reasons and lack of education.^11^

No comprehensive studies concerning the oral health habits and denture hygiene of denture wearers in Romania are currently available. The present study investigated denture wearers from Central Transylvania, Romania, with respect to their sociodemographic characteristics, health status, oral hygiene habits, and their denture hygiene status. The study group included mainly elderly patients (69.1% over 60 years of age), with 81% of all patients reporting dental caries as the main cause of tooth loss. The same trend was reported in previous research^4^ regarding the prevalence of edentulism in young adults in Romania and the association with the presence of dental caries. Poor oral health status among young people was found to be associated with low public expenditure on oral health as well as insufficient prevention programmes (in recent years, these received as little as 0.6% of the public-sector budget for oral healthcare^31^). Moreover, according to the National Institute of Statistics, the number of inhabitants per dentist was six times higher in rural than in urban areas in 2017.

Poor denture hygiene is associated with a series of local and systemic conditions, making it a serious public health issue. The denture wearers included in the present study reported a series of associated diseases, among which cardiovascular disease was most prevalent (51.2%), slightly higher than the results of a study on patients from another historical region of Romania (Moldavia, 42.2%).^17^ When masticatory function is affected, eating habits change, as rough, fibrous food is avoided or eliminated and replaced with food rich in saturated fats and cholesterol,^28,33^ which are associated with cardiovascular disease.

Experimental studies showed that covering the palatal mucosa with the acrylic material favoured the inflammatory response of the underlying tissues.^5^ The mucosal surface of the denture is considered to be one of the main reservoirs of various microbes.^8,42^ After clinical assessment of denture hygiene by analysing the internal surface, only half of the dentures (52.4%) in the study group exhibited good or optimal hygiene status. Although research shows that the internal surface of the denture is more exposed to microbial colonisation than the palatal mucosa,^19,35^ it seems that denture wearers are less concerned about their oral hygiene than patients who still have teeth.^39^ Moreover, studies show that patients tend to overestimate the efficacy of their hygiene measures, since the level of denture cleanliness was usually low even for patients who reported regular cleaning practices.^15^ Studies in other European countries show that denture wearers clean their dentures at least once a day.^9,29^ More than half of the subjects in the present study reported denture cleaning 2-3 times a day, but only a low percentage of the dentures were found to have an optimal hygiene status, showing that the responses could have been overestimated. The results showed denture hygiene status was not correlated with their answers regarding oral hygiene habits. Schou et al^36^ also found no relation between denture plaque and brushing habits.

Some authors suggested that cleaning frequency could be related to the ability to brush (sometimes considered difficult by elderly people) and remembering to brush dentures.^30^ In our study group, the participants reported no difficulties with the cleaning procedures; most of the patients (94%) perceived them as easy (Table 2).

**Table 2:** Difficulty of denture cleaning

Variable	n	%
Difficult	9	5.56
Easy	153	94.44

n=number of patients.

Our results revealed a strong connection between denture cleaning frequency and gender. As other European researchers previously reported,^3,9,29^ we also noted a significantly higher frequency of oral hygiene procedures among women than men, in all age groups.

A study that analysed recommendations by dental health professionals showed that dentists who instruct their patients at the time of denture delivery usually advise them to combine two cleaning methods: denture cleansing tablets and brushing with toothpaste were their first choices.^2^ Proper instructions regarding complete denture wearing are sometimes neglected. Although most of the dentists included in a survey (83%) admitted the importance of patient education on effective denture cleaning, 48% found instructing the elderly patients to be time-consuming; 70% of them reported using verbal instructions, while only 14% provided practical demonstrations.^41^ On the other hand, studies that included denture wearers revealed that 77.5%^26^ and 82.9%^12^ were not instructed at all at denture delivery. Hong et al^20^ showed that more than half of the dentists had limited information on denture cleaning products and 87% admitted needing to improve their knowledge on that topic.

Although denture cleaning with soap and non-abrasive cleansers at least once a day are highly recommended, in the current study, the most frequently reported method was brushing with a toothbrush and toothpaste. This was the second cleaning method preferred by dentists, recommended by 46.6% of them, after immersion of the dentures in solutions of commercial cleansing tablets (71.5%).^12^ Sheen et al^39^ showed a significant reduction in visual plaque for dentures soaked in a denture cleanser. Duyck et al^14^ showed that denture storage in water containing a dissolved disinfectant tablet reduced the number of bacteria on dentures and the biofilm mass, but did not seem to have any effect on *Candida* colonisation. *Candida* infection appears to be one of the etiological factors of stomatitis, a condition frequently associated with poor oral hygiene and also with continuous wearing of dentures. Of our respondents, only 30.9% always removed their dentures overnight. This is in contrast to Baran et al,^3^ who found 44.8% for overnight denture removal. The present values were higher than those reported by Marchini et al (26.3%).^26^

Considering the uneven geographic distribution of medical personnel in Romania and the numerous patients who do not have continuous access to professional dental care,^13^ the implementation of effective prophylactic and therapeutic measures for patients in both urban and rural areas is essential. A coherent strategy requires prior knowledge of the patients’ profile and habits regarding oral care; thus, our study could be of use to reveal denture wearers’ hygiene misunderstandings and mistakes, as well as points where improvement is needed. Our study – to our knowledge, the first one on this topic carried out in Romania – assessed the influence of the sociodemographic characteristics and health status on the oral hygiene habits and denture hygiene status of complete denture wearers living in Transylvania.

Following this study, a documented intervention could start with raising the awareness of health professionals regarding the groups of patients at risk, in addition to reaching a consensus regarding the most effective oral hygiene procedures for denture wearers. Next, patients and their caregivers could be informed about the most suitable methods of denture cleaning, their recommended frequency, and also overnight removal and storage of the dentures. Appropriate instructions at the time of denture delivery, together with periodic recalls for reinforcement of denture care instructions and oral health and denture status evaluation are highly important for complete denture users.

### Limitations

The use of questionnaires, even if anonymous, is error prone, as questions can be misunderstood (especially by older patients), or deliberately answered falsely, especially when asking about personal health status or hygiene habits. The final results could thus be inaccurate. The participants enrolled in the research were recruited and filled in the questionnaires at their dental visit. However, a group of denture wearers were disregarded by our study: those who were unable to travel because of old age or poor health status, or those who did not visit dental practitioners. Subsequent studies can overcome this limitation by collecting data from patients institutionalised in residential homes, and by including more participants, which will enable the analysis of a more representative sample.

### Practical Recommendations

The recommendations of this study are directed at all those involved in the complex problem of poor oral health of denture wearers. Education about oral hygiene, better access to fluoridated water and secondary care even in rural areas can prevent tooth loss.^11^ Dentists could be enrolled in continuous medical training programmes on the topic of denture hygiene, new methods and products for denture cleaning, in order to consistently inform and advise patients. Effective recommendations, especially for the elderly and their caregivers, could be associated with practical demonstrations and written instructions. The geriatric population should be given proper attention regarding both therapeutic and prophylactic measures. Moreover, patients of all ages should be encouraged to increase the frequency of their dental visits. The early diagnosis and treatment of dental caries and periodontal lesions should be a priority for both patients and dentists, in order to avoid tooth loss.

## Conclusion

The need for geriatric oral health care will increase in the coming years, due to the continuous rise in life expectancy. Oral health is an important part of general health and plays an important role in denture wearers’ quality of life. Besides oral rehabilitation, the dentists should support denture wearers in terms of denture and oral hygiene, through proper measures such as specific recommendations, instructions and periodic recalls.

## Questionnaire

1. Your age
  - lower than 40
  - between 41 and 50
  - between 61 and 70
  - more than 71
2. Gender
  - female
  - male
3. Professional activity
  - active
  - retired
4. Residence
  - urban
  - rural
5. Smoker
  - yes
  - no
6. General diseases
  - cardiovascular diseases
  - hepatic diseases
  - renal diseases
  - digestive diseases
  - endocrine diseases
  - bone diseases
  - dermatologic diseases
  - allergic diseases
  - other diseases – mention them
7. You wear
  - upper denture
  - lower denture
  - both
8. Cause of tooth loss
  - caries
  - periodontal diseases
  - traumatic
9. You wear the denture overnight
  - yes
  - no
  - sometimes
10. How often do you clean your denture?
  - 2–3 times per day
  - once per day
  - once every few days
11. What products do you use for denture cleaning?
  - toothpaste
  - commercial tablets
  - soap
  - vinegar mixed with water
  - other products - mention them
12. What instruments do you use
  - toothbrush
  - brush (similar to nail brush)
  - I don’t use a brush
13. You find denture cleaning
  - easy
  - quite difficult
  - very difficult
